# The Role of Fluorescence In Situ Hybridization in Pancreatobiliary Brushing Cytology: A Large Retrospective Review with Histologic Correlation

**DOI:** 10.3390/diagnostics12102486

**Published:** 2022-10-14

**Authors:** Jaffar Khan, Carlo De la Sancha, Mohammed Saad, Ahmad Alkashash, Asad Ullah, Fatimah Alruwaii, Luis Velasquez Zarate, Harvey M. Cramer, Howard H. Wu

**Affiliations:** 1 Department of Pathology and Laboratory Medicine, Indiana University School of Medicine, Indianapolis, IN 46202, USA; 2 Department of Pathology and Laboratory Medicine, Vanderbilt University, Nashville, TN 37232, USA; 3 Department of Pathology and Laboratory Medicine, Henry Ford Health, Detroit, MI 48202, USA; 4 Department of Pathology and Laboratory Medicine, Augusta University School of Medicine, Augusta, GA 30912, USA

**Keywords:** bile duct brushing, cytology, FISH, pancreaticobiliary tract

## Abstract

(1) Background: Although the specificity of brush cytology for the detection of malignant pancreaticobiliary strictures is high, its sensitivity is low. Fluorescence in situ hybridization (FISH) can be used to detect chromosomal aneuploidy in biliary brushing specimens, and when used as an adjunct to routine cytology, it significantly improves diagnostic sensitivity. (2) Methods: We searched our laboratory information system to identify all bile duct brush cytology cases with follow-up surgical pathology between January 2001 and September 2019. Cytologic diagnoses were classified as negative, atypical, suspicious, or malignant. Correlated surgical pathological diagnoses were classified as benign or malignant. FISH test results were obtained for a subset of cytology cases with concurrent FISH testing, and the sensitivity, specificity, positive predictive value, and negative predictive value in identifying malignancy for cytology alone, FISH alone, and combined cytology and FISH were calculated. (3) Results: A total of 1017 brushing cytology cases with histologic correlation were identified. A total of 193 FISH tests were performed concurrently with cytological specimens. Malignant diagnoses were identified in 623 of 1017 patients, while 394 patients had benign strictures. The sensitivity, specificity, positive predictive, and negative predictive rate were 65%, 78%, 83%, and 49% for cytology alone; 72%, 67%, 63%, and 68% for FISH alone; and 85%, 42%, 60%, and 74% for combined cytology and FISH, respectively. Among FISH-positive cases, the risk of malignancy for polysomy was 82% and 32% for trisomy. (4) Conclusions: FISH improves the sensitivity and negative predictive rate of bile duct brush cytology. The combination of cytology and FISH has increased the sensitivity from 65% to 85% and the negative predictive rate from 49% to 74% when compared to cytology alone. A patient with a polysomy FISH result had a significantly higher risk of malignancy than a patient with a trisomy 7 result (82% vs. 32%, *p* < 0.00001).

## 1. Introduction

Differentiating between benign and malignant biliary strictures is very challenging [1]. Malignant bile duct strictures are commonly caused by cholangiocarcinomas, periampullary cancers, and pancreatic cancers. Most of these cancers are diagnosed at advanced stages and are usually unresectable [1,2,3]. The prognosis of these patients may improve with earlier diagnosis and treatment [4]. Surgical resection is sometimes associated with a high rate of postoperative morbidity [5,6,7]. Fluorescence in situ hybridization (FISH), originally developed in the 1980s, is an adjunctive test that is helpful in confirming the presence of putative malignant cells in brush cytology samples [8]. Since approximately 7–10% of patients undergoing surgery for suspected extrahepatic biliary malignancies are found to have benign pathology, confirming the presence of a malignant diagnosis through preoperative evaluation of cytologic samples and/or surgical biopsy is essential before considering aggressive surgical management [9]. The most common procedure used to evaluate suspicious biliary strictures is endoscopic retrograde cholangiopancreatography (ERCP), as it is easier to perform and is associated with fewer and less severe adverse events than surgery. Malignancy can be diagnosed using brush cytology or intraductal biopsy [10].

## 2. Methods and Material

An electronic search of our department’s cytology records was performed to identify all bile duct brushing cytology cases with correlating biopsy, resection, and FISH testing that occurred between January 2001 and September 2019. Cytologic diagnoses were classified as negative, atypical, suspicious, or malignant. These results were correlated with subsequent surgical pathology diagnoses, which were classified as either benign or malignant. Correlation studies were performed between cytology and histology, FISH and histology, and a combination of cytology/FISH and histology. To simplify the statistical analysis, cytological diagnoses of ‘atypical’, ‘suspicious’, and ‘malignant’ were regarded as’positive’. The sensitivity, specificity, positive predictive value (PPV), and negative predictive value (NPV) were calculated. Statistical analysis was performed using the chi-squared test (https://www.socscistatistics.com/tests/chisquare2/default2.aspx) accessed on 1 February 2022.

## 3. Results

A total of 1017 cases with both cytology and correlating biopsy and/or resection results were retrieved. Cytologic diagnoses included: negative 518 (51%), atypical 235 (23%), suspicious 91 (9%), malignant 158 (16%) and nondiagnostic 15 (1%). Of these, 623 patients (61%) were diagnosed with malignancy and 394 patients (39%) had benign diagnoses on correlating biopsy or resection. Of the 518 cytologically negative cases, 305 were benign (59%) and 213 (41%) were malignant on follow-up histology. The follow-up histological diagnoses for each category are shown in Table 1. FISH was performed on 193 cytological specimens (19%). FISH showed abnormalities in 112 samples (58%), including 68 polysomy (61%) and 44 trisomy (39%), while 81 samples ((42%) were negative. Of the 81 FISH-negative cases, 27 (33%) proved to be malignant and 54 (6%) benign on final histopathology. For FISH-positive cases (*n* = 112), 70 patients (63%) were diagnosed with malignancy and 42 (37%) were diagnosed as benign (Table 2).

For statistical analysis, we have grouped cytologic diagnoses of atypia, suspicious and malignant as “positive”, the sensitivity, specificity, PPV and NPV in identifying malignancy based on cytology alone were 65%, 78%, 83% and 49%. The sensitivity, specificity, PPV, and NPV for identifying malignancy based on FISH alone were 72%, 67%, 63%, and 68%, respectively. If we combined cytology and FISH, the sensitivity, specificity, PPV, and NPV in identifying malignancy would be 85%, 42%, 60%, and 74%, respectively. (Table 3 and Table 4).

Of the 68 FISH positive polysomy cases, 56 were malignant and 12 were benign. Out of 44 FISH positive trisomy 7 cases, 14 were malignant and the remaining 30 showed benign pathology. The risk of malignancy in polysomy cases was significantly higher than that in trisomy cases (82% vs. 32%, *p* < 0.00001) (Table 5).

FISH findings and related diagnoses for each cytologic category of negative and atypia, which showed polysomy FISH results were significantly more associated with malignancy than negative and trisomy FISH results (*p* < 0.05, Table 6a,b). In the suspicious cytology category, the risk of malignancy is 92%. Of the malignant cases with FISH performed in the suspicious category, FISH polysomy was noted in 75% (12/16) (Table 6c). In the malignant cytology category, the risk of malignancy is 99%. Of the malignant cases with FISH performed in malignant category, FISH polysomy was noted in 74% (17/23) of cases. (Table 6c).

FISH was not performed in two false-positive cases. For these two cases, the follow-up histology were biopsies. For the first case, the histologic diagnosis was “Small fragments of normal-adenoma with high-grade dysplasia”. Both patients were treated with radiation therapy and were lost to follow-up within six months. The radiographic impression for these two cases were “malignant appearing”. Although the histological diagnoses were not invasive carcinomas, these two cases were probably malignant. These two cases most likely represent false-positive cases”. The specificity of malignant diagnosis based on brush cytology was nearly 100%.

## 4. Discussion

All patients with suspected extrahepatic biliary, ampullary, and pancreatic head malignancies were evaluated using endoscopic retrograde cholangiopancreatography (ERCP) to accurately define biliary and pancreatic anatomy. As an integral component of the ERCP procedure, various biopsy sampling techniques, including brush cytology, forceps biopsy, and fine needle aspiration, are routinely used to establish definitive and confirmatory pathological diagnoses. Unfortunately, the sensitivity of the above-mentioned diagnostic tests for detecting malignancy is low [11,12,13]. The most commonly used technique is brush cytology, with a high specificity ranging from 80% to100%, but low sensitivity, ranging from 20% to 45% [14,15].

Primary sclerosing cholangitis (PSC) is a chronic cholestatic liver disease associated with a high risk of cholangiocarcinoma. Screening and surveillance of patients with PSC for cholangiocarcinoma using ERCP combined with cytology have the greatest potential impact on patient survival. To improve the yield of conventional cytology, fluorescence in situ hybridization (FISH) has been used to detect chromosomal abnormalities associated with malignancies. FISH utilizes complementary DNA probes to detect abnormal cells in cytological specimens. FISH detects chromosomal changes that have been described in approximately 80% of malignant biliary neoplasms. According to some studies, the reported sensitivity of FISH for the detection of malignancies ranges from 50% to 60% [16,17,18]. Fluorescent-labeled probes that hybridize with the nuclear DNA of individual cells are utilized in FISH to detect chromosomal changes in samples obtained from the brushing of biliary strictures. Polysomy of chromosomes 3, 7, and 17 along with deletion of chromosome locus 9p21 can be detected by FISH probes. These genetic abnormalities can be found in many epithelial cancers, including those affecting the biliary epithelium. The addition of FISH to routine brush cytology can be valuable in evaluating biliary strictures [19,20,21,22,23].

The primary advantage of supplementing cytology with FISH analysis is an increase in the sensitivity of cholangiocarcinoma detection without reducing specificity. FISH can also help to define lesions that are considered suspicious for malignancy by cytology, positive FISH result in indeterminate biliary stricture case would support a malignant diagnosis while FISH being negative will favor benign stricture. Prior stenting can be a limiting factor in biliary brush cytology, as reactive atypia can resemble carcinoma in this setting and pose a challenge to render the correct diagnosis. Due to the cytomorphological overlap, some pathologists are reluctant to make a malignant diagnosis in patients with recent history of stenting [24].

To the best of our knowledge, this single-institution retrospective review of 1017 biliary brush cytology cases with histologic correlation with a significant subset of FISH analyses is the largest such review reported. This is perhaps comparable with the review article of Kipp et al. [25], which analyzed data from 13 publications that encompassed 1832 patients (ranging from 1 to 498 patients per study). Kipp et al. demonstrated an improvement in sensitivity when combining routine cytology and FISH in comparison to cytology assessment alone; for example, in 2003, their group evaluated 131 patients and determined that the sensitivity was greater when using FISH (34% vs. 15%) compared to cytology alone. Another example was a study of 498 patients by Fritcher, who demonstrated that the addition of FISH significantly increased the diagnostic sensitivity from 20% to 40% compared with cytology alone.

In a prospective study performed between September 2008 and August 2010) Smoczynski et al. [19] studied 81 patients with bile duct or pancreatic duct strictures. When atypia was identified as positive, the resultant sensitivity was 53.7% and the sensitivity of FISH was 51.85%. The sensitivity improved to 72.22% when either cytology with atypia was positive or when the FISH result was positive. Our study, with 1017 cytology and 193 FISH results, showed a similar result demonstrating a sensitivity of 65% for cytology alone and 72% for FISH alone. The sensitivity significantly improved to 85% for combined cytology and FISH.

A recent study by Brooks et al. [26] analyzed the importance of adding FISH to routine cytology for the evaluation of pancreatic duct strictures. Brooks et al. presented a large study of 281 patients with pancreatic duct strictures who underwent FISH and compared them with routine cytology and cholangioscopic biopsy. They reported that routine brush cytology had a sensitivity of 35% when it included the presence of suspicious or malignant cells, and the sensitivity improved to 55% when they included both polysomy and loci 9p21 heterozygous deletions as positive results. In comparison, our study included atypia as positive and resulted in 65% sensitivity, which increased to 85% with the addition of the FISH test.

Fritcher et al. published the most comprehensive study, including 498 patients with cytology, and FISH testing demonstrated that the sensitivity of FISH for detecting malignancy was significantly higher than that of cytology (43% vs. 20%). This study also indicated that a patient with a polysomy FISH result had a 77.6 times higher chance of having malignancy than a patient with a negative FISH result, and their study also suggested that a patient with a polysomy FISH result was at a higher risk of malignancy than a patient with a trisomy 7 result (98% vs. 48%) [27]. In our study, we also verified that a patient with polysomy FISH results had a risk of malignancy of 82%, which is significantly higher than the risk of malignancy (32%) in a patient with trisomy 7 FISH results (*p* < 0.00001). Although the specificity of cytological diagnosis is very high, it is not the same for FISH according to our study. Next-generation sequencing can be used as an adjunct to cytologic evaluation for both increasing the sensitivity of pathologic workup and identifying potential targetable alterations in limited but valuable cytology specimens [28]. The diagnosis for biliary duct strictures still remains a challenge. Studies suggest if ERCP for tissue sampling does not yield satisfactory diagnostic result, the next best option is to use Chol angioscopy and Endoscopic ultrasound [29].

Our study intends to persuade pathologists and other healthcare workers to routinely add FISH to the cytology assessment when pancreatic and biliary tract neoplasms are within the scope of clinical differential diagnosis. Our study showed that 92% of the cases that were suspicious on cytology proved to be malignant on subsequent biopsy; therefore, the diagnosis suspicious for malignancy should make the clinical team approach the patients with aggressive management. If we can increase the sensitivity and negative predictive value of our diagnostic tools, we may help avoid unnecessary total or partial pancreatectomies and be more confident in treating a subset of these patients in a more conservative manner. The value of long-term outcome analysis, as shown in this report, is significant because, as we highlighted earlier, approximately 10% of surgeries performed for diagnostic purposes proved to be benign.

## 5. Conclusions

With 1017 patients, this is the largest biliary brush cytologic study with follow-up histologic correlation. Our study showed that the addition of FISH significantly improved the diagnostic sensitivity (85% vs. 65%) and negative predictive rate (74% vs. 49%) compared with bile duct brushing cytology alone for detecting malignancy. Our study also demonstrated that a patient with polysomy FISH results was associated with a significantly higher risk of malignancy than a patient with trisomy 7 results (82% vs. 32%, *p* < 0.00001).

## Figures and Tables

**Table 1:** Correlation of cytological subcategory diagnoses and final surgical pathology diagnoses.

Surgical Dx	Negative	Atypia	Suspicious	Malignant	Non-Diagnostic
Benign *n* = 394	305	75	7	2	5
Malignant *n* = 623	213	160	84	156	10
Total *n* = 1017	518	235	91	158	15

**Table 2:** Correlation of FISH and final surgical pathology diagnoses.

Surgical Dx	FISH Negative	FISH Positive
Benign	54	42
Malignant	27	70

**Table 3:** Correlation of combined cytology with addition of FISH and final surgical pathology diagnoses.

Surgical Pathology Diagnosis	Negative (Both Cytology and FISH Negative)	Positive (Either Cytology Positive or FISH Positive)
Benign	42	54
Malignant	15	82

**Table 4:** Comparison of sensitivity, specificity, PPV and NPV.

	Cytology	FISH	Cytology + FISH
Sensitivity	65%	72%	85%
Specificity	78%	67%	42%
Positive predictive value	83%	63%	60%
Negative predictive value	49%	68%	74%

**Table 5:** Correlation of FISH-positive cases and final surgical pathology diagnoses.

Surgical Pathology Diagnosis	FISH Polysomy	FISH Trisomy
Benign	12	30
Malignant	56 (82%)	14 (32%)

**Table 6:** (**a**) Correlation of cytology-negative cases with FISH and histological diagnosis. (**b**) Correlation of cytological atypia cases with FISH and histological diagnosis. (**c**) Correlation of suspicious cytology cases with FISH and histological diagnosis. (**d**) Correlation of cytological malignant cases with FISH and histological diagnosis. (**e**) Correlation of cytology non-diagnostic cases with FISH and histological diagnosis.

(**a**)								
**Cytology FISH Result**		**Histology Benign**		**Histology Malignant**		**Total Number**		**Chi-Square**
Negative		42		15		57		
Polysomy		8		14		22		*p* = 0.002
Trisomy		20		5		25		*p* = 0.5
Not performed		235		179		414		
Total		305		213		518		
(**b**)								
**Cytology FISH Result**	**Histology Benign**			**Histology Malignant**		**Total Number**		**Chi-Square**
Negative	12			6		18		
Polysomy	3			13		16		*p* = 0.005
Trisomy	10			4		14		*p* = 0.7
Not performed	50			137		187		
Total	75			159		235		
(**c**)								
**Cytology FISH Result**			**Histology Benign**		**Histology Malignant**		**Total Number**	
Negative			0		2		2	
Polysomy			1		12		13	
Trisomy			0		2		2	
Not performed			6		68		74	
Total			7		84		91	
(**d**)								
**Cytology FISH Result**			**Histology Benign**		**Histology Malignant**		**Total Number**	
Negative			0		4		4	
Polysomy			0		17		17	
Trisomy			0		2		2	
Not performed			2		133		135	
Total			2		156		158	
(**e**)								
**Cytology FISH Result**			**Histology Benign**		**Histology Malignant**		**Total Number**	
Negative			0		0		0	
Polysomy			0		0		0	
Trisomy			0		1		1	
Not performed			5		9		14	
Total			5		10		15	

## Data Availability

Data are available on request due to restrictions, e.g., privacy or ethical.
